# Detection of homogentisic acid by electrospray ionization mass spectrometry

**DOI:** 10.1002/jcla.24976

**Published:** 2023-10-25

**Authors:** Yasunori Tokuhara, Kazuaki Ohara, Tatsuya Morinishi, Kentaro Yamaguchi, Satoshi Tada

**Affiliations:** ^1^ Department of Medical Technology Kagawa Prefectural University of Health Sciences Takamatsu Kagawa Japan; ^2^ Faculty of Pharmaceutical Sciences at Kagawa Campus Tokushima Bunri University Sanuki Kagawa Japan

**Keywords:** alkaptonuria, ascorbic acid, electrospray ionization mass spectrometry, homogentisic acid, pH

## Abstract

**Objective:**

Homogentisic acid (HGA) is excreted in excessive amounts in the urine of patients with alkaptonuria, which is a hereditary metabolic disorder of phenylalanine and tyrosine. Therefore, the detection of HGA in urine is useful for the diagnosis of alkaptonuria. To evaluate the detection of HGA, we confirmed the color shift of HGA solutions and analyzed them by electrospray ionization mass spectrometry (ESI‐MS).

**Methods:**

We observed the color change of the HGA solutions under different pH conditions (pH 6.0, 7.0, and 8.0) and examined the influences of adding potassium hydroxide (KOH) and ascorbic acid (AA) to the HGA solutions. Then, we analyzed the chemical reaction in HGA solutions using ESI‐MS.

**Results:**

The HGA solution at pH 8.0 became brown after incubation at room temperature for 24 h and became darker brown with the addition of KOH; however, HGA solutions at pH 6.0 and 7.0 showed no color changes. The brown color change of the HGA solution at pH 8.0 was also inhibited by AA. Moreover, all HGA sample solutions showed the deprotonated molecular ion peak at m/z 167.035 in the negative ion mode after incubation at room temperature for 24 h and with the addition of KOH and AA.

**Conclusion:**

We identified the molecular ion of HGA in all sample solutions by ESI‐MS, regardless of different pH conditions, color changes, or the presence of AA. These results suggest that spectral analysis by ESI‐MS is suitable for the detection of HGA and the diagnosis of alkaptonuria.

## Graphical abstract

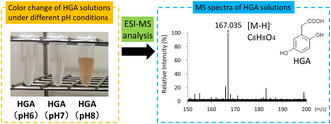

## 1 INTRODUCTION

**FIGURE 1:**
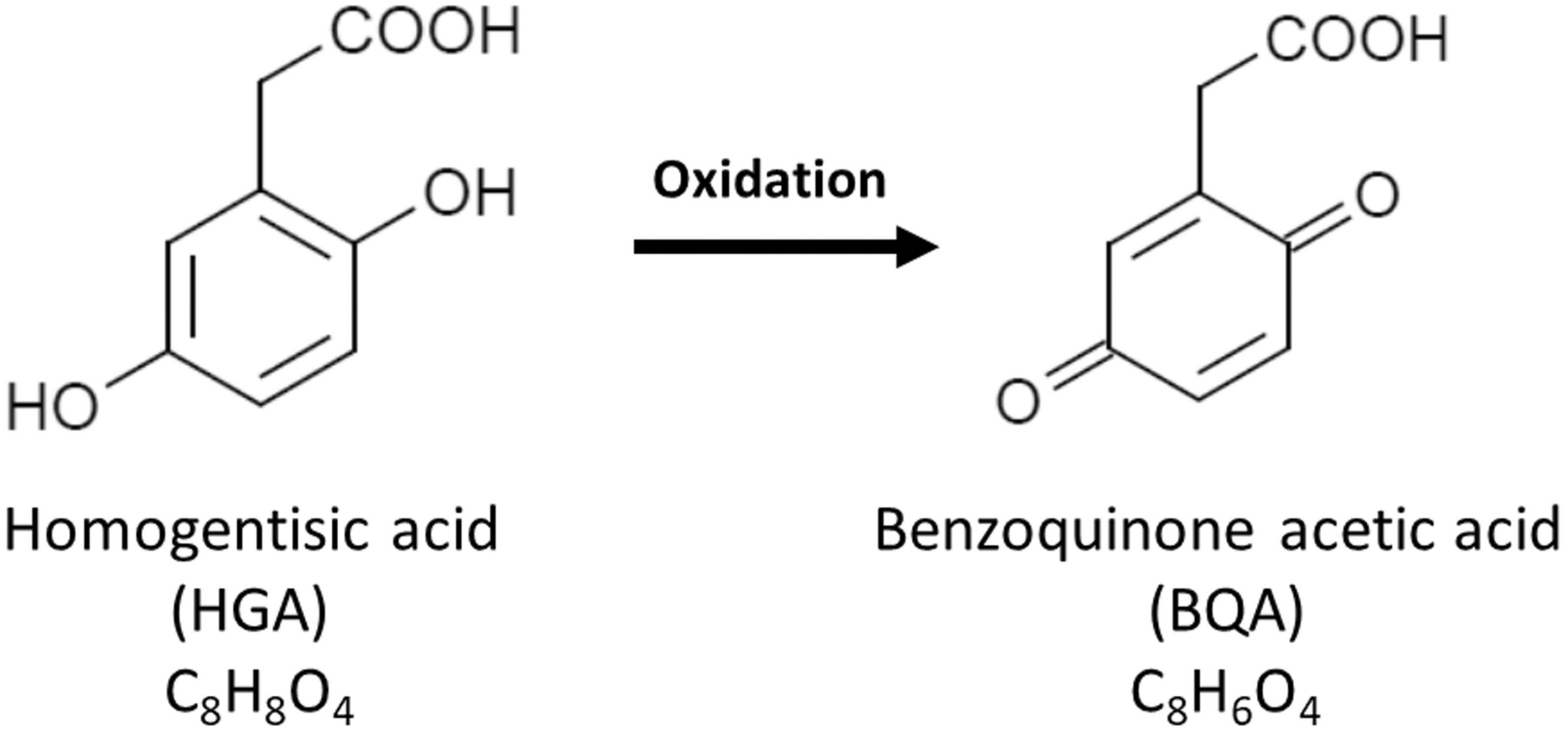
Structures of homogentisic acid (HGA) and benzoquinone acetic acid (BQA).

Alkaptonuria is a hereditary metabolic disorder of phenylalanine and tyrosine caused by a deficiency of enzyme homogentisic acid (HGA) oxidase.^1^, ^2^ This defect leads to the accumulation of HGA in connective tissue leading to ochronosis, and excessive amounts of HGA are excreted in the urine of patients with alkaptonuria.^3^, ^4^ Alkaptonuric urine turns dark brown at room temperature after several hours to days owing to the oxidation of HGA to benzoquinone acetic acid (BQA)^5^, ^6^, ^7^ (Figure 1). Moreover, the oxidation of HGA to BQA is accelerated under an alkaline pH.^8^, ^9^, ^10^

We have previously reported that the oxidation reaction of HGA to BQA was accelerated by the addition of sodium hydroxide (NaOH) and sodium hypochlorite pentahydrate (NaOCl·5H_2_O), which is a strong oxidant.^11^ In addition, we observed that alkaptonuric urine or HGA solution became dark brown and showed characteristic absorbance peaks following the addition of NaOH and NaOCl·5H_2_O.^11^ We have also reported that the oxidation of HGA and brown color change that occur after the addition of NaOH and NaOCl·5H_2_O were inhibited by ascorbic acid (AA), which is an antioxidant.^11^, ^12^

In the present study, we observed the color changes of HGA solutions under different pH conditions and examined the influences of adding alkaline solution and AA on the oxidation of HGA. Furthermore, we analyzed the HGA solutions by electrospray ionization mass spectrometry (ESI‐MS). We identified the molecular ion of HGA in all sample solutions by ESI‐MS, regardless of different pH conditions, color changes, or the presence of AA. Thus, this method enables the reliable detection of HGA in urine samples obtained from patients with alkaptonuria.

## 2 MATERIALS AND METHODS

### 2.1 Reagents

Homogentisic acid was purchased from Tokyo Chemical Industry Co., Ltd. (Tokyo, Japan). AA, 0.5 M potassium hydroxide (KOH), and 0.1 mol/L phosphate buffer solution (pH 6.0, 7.0, or 8.0) were purchased from Wako Pure Chemical Industries, Ltd. (Osaka, Japan).

### 2.2 Sample preparation and observation of the color change

A solution of 10.0 g/L HGA was prepared from high‐purity analytical reagents and pure water. Then, a total of 800 μL of each 1.0 g/L HGA solution (pH 6.0, 7.0, and 8.0) was prepared by mixing 80 μL of 10.0 g/L HGA and 720 μL of pH 6.0, pH 7.0, or pH 8.0 phosphate buffer solution, respectively. Next, these samples were incubated at room temperature for 24 h. For the addition of the alkaline solution, 30 μL of 0.5 M KOH was added to 800 μL of 1.0 g/L HGA (pH 6.0, 7.0, and 8.0), and these samples were incubated at room temperature for 1 h. To examine the effects of an antioxidant, 40 μL of 10.0 g/L AA was added to the mixture of 80 μL of 10.0 g/L HGA and 680 μL of pH 6.0, pH 7.0, or pH 8.0 phosphate buffer solution, followed by the addition of 30 μL of 0.5 M KOH, and these samples were also incubated at room temperature for 1 h.

### 2.3 Electrospray ionization mass spectrometry (ESI‐MS)

The HGA solutions were measured by Fourier transform ion cyclotron resonance mass spectrometry using SolariX 9.4 T (Bruker Daltonics, Bremen, Germany). Mass spectra were calibrated using external calibration with a tuning‐mix (Agilent, Santa Clara, CA, USA). The following instrument parameters were used: the sample flow rate was 2 μL/min, the desolvation plate temperature was 150°C, the rate of N_2_ drying gas was 2.5 L/min, the rate of N_2_ nebulizing gas was 1.5 L/min, and the capillary voltage was 4.5 kV for the negative ion detection mode.

## 3 RESULTS

### 3.1 Color changes of HGA solutions under different pH conditions

**FIGURE 2:**
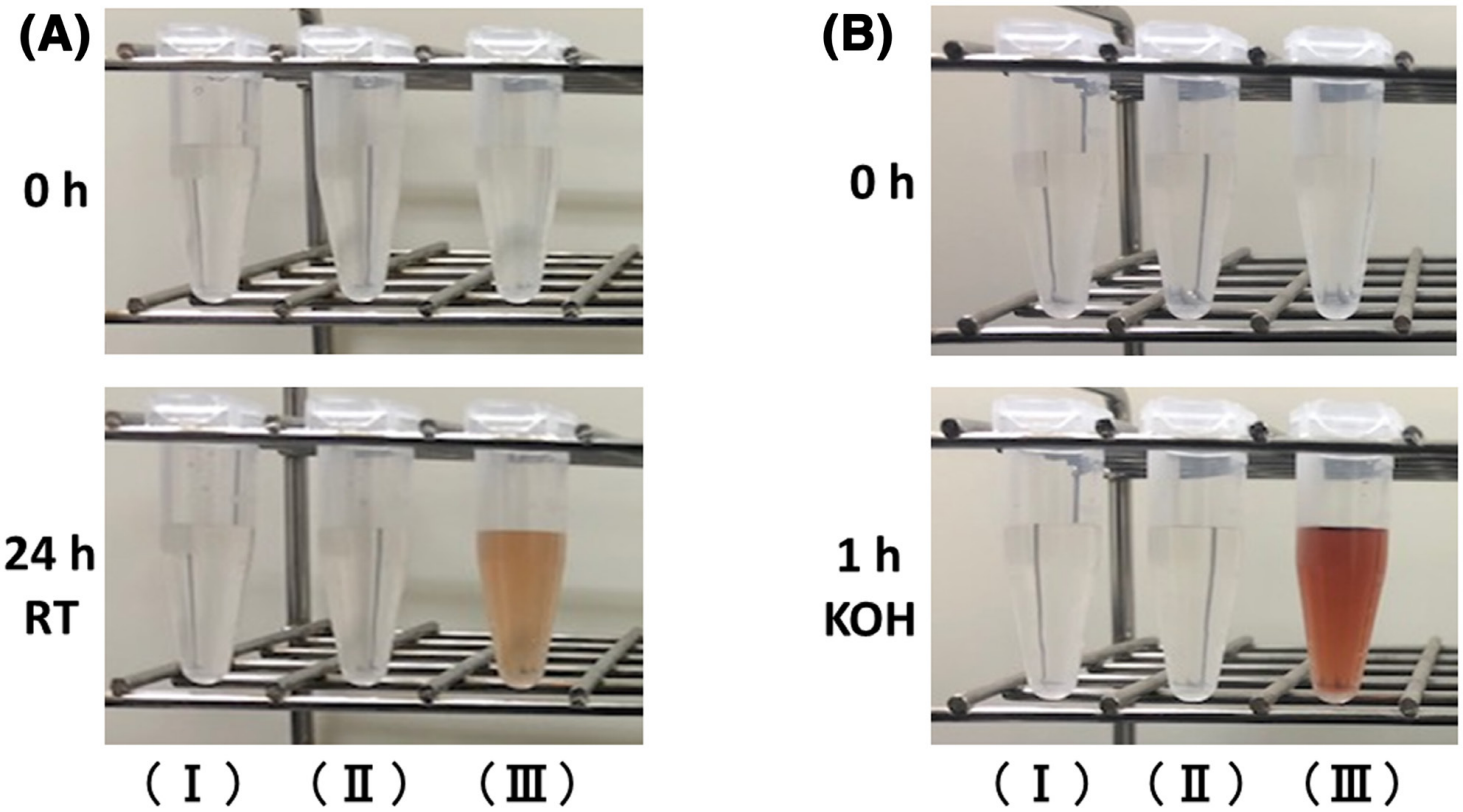
Color change of HGA solutions. (A) HGA solutions at pH 6.0 (I), pH 7.0 (II), and pH 8.0 (III) after incubation at room temperature for 24 h. (B) HGA solutions at pH 6.0 (I), pH 7.0 (II), and pH 8.0 (III) after incubation with KOH at room temperature for 1 h.

First, we observed the color changes of the HGA solutions under different pH conditions (pH 6.0, 7.0, and 8.0). The HGA solution at pH 8.0 became brown after incubation at room temperature for 24 h (Figure 2A). In contrast, the HGA solutions at pH 6.0 and 7.0 were transparent and color changes were not recognized before or after incubation at room temperature for 24 h (Figure 2A). Next, we added the KOH, which is a strong alkali, to the HGA solutions (pH 6.0, 7.0, and 8.0) and observed the color changes. The HGA solution at pH 8.0 became dark brown after the addition of KOH (Figure 2B). However, the HGA solutions at pH 6.0 and 7.0 incubated with KOH did not show color changes (Figure 2B).

### 3.2 Effect of ascorbic acid

**FIGURE 3:**
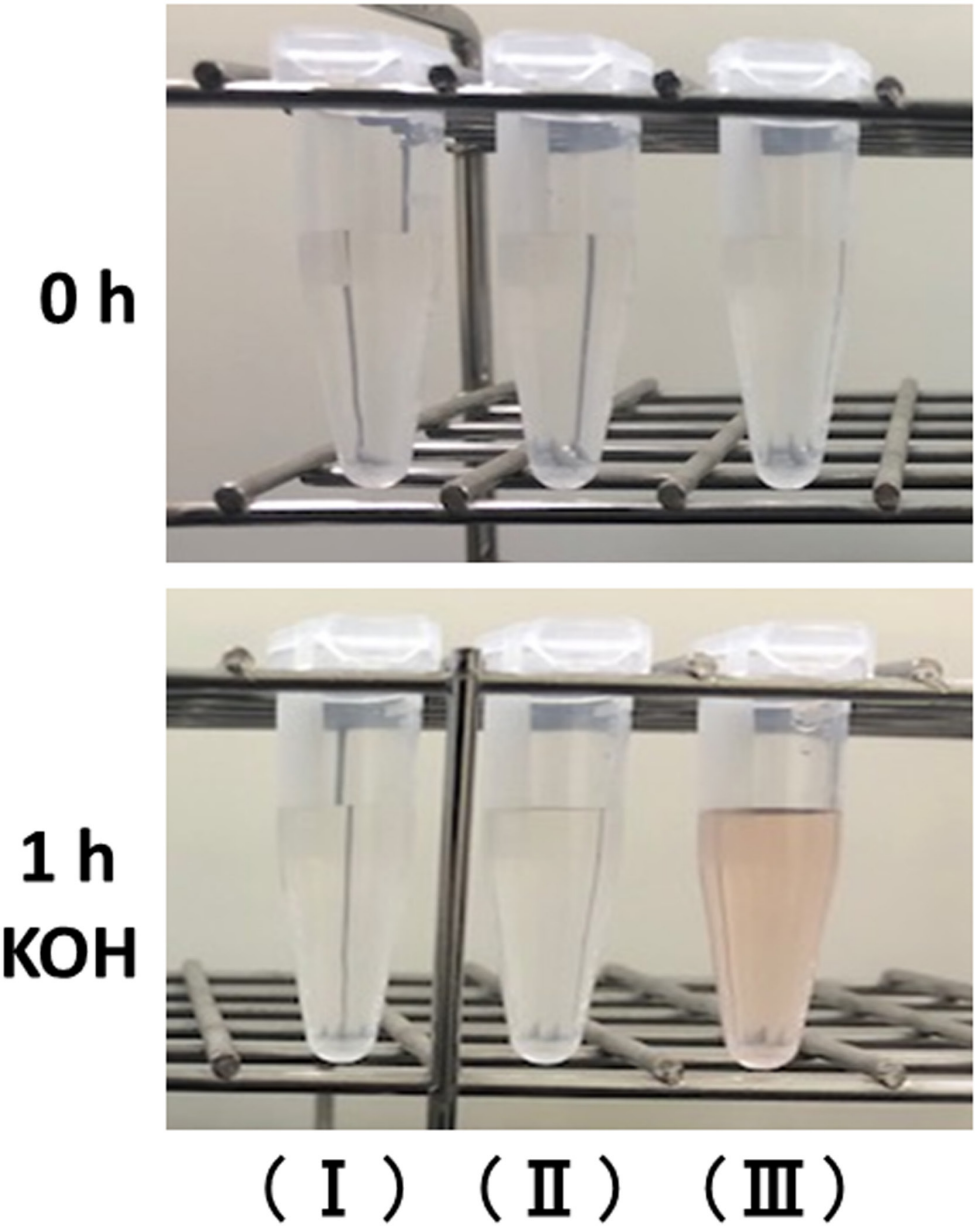
Color change of HGA solutions treated with AA. AA was added to the HGA solutions at pH 6.0 (I), pH 7.0 (II), and pH 8.0 (III), and then KOH was added to each sample, followed by incubation of the samples at room temperature for 1 h.

We then investigated whether AA, an antioxidant, affected the color changes of HGA solutions (pH 6.0, 7.0, and 8.0). The HGA solution at pH 8.0 treated with AA turned a light brown color after the addition of KOH (Figure 3). Conversely, the HGA solutions at pH 6.0 and 7.0 treated with AA did not show color changes (Figure 3).

### 3.3 ESI‐MS analysis

**FIGURE 4:**
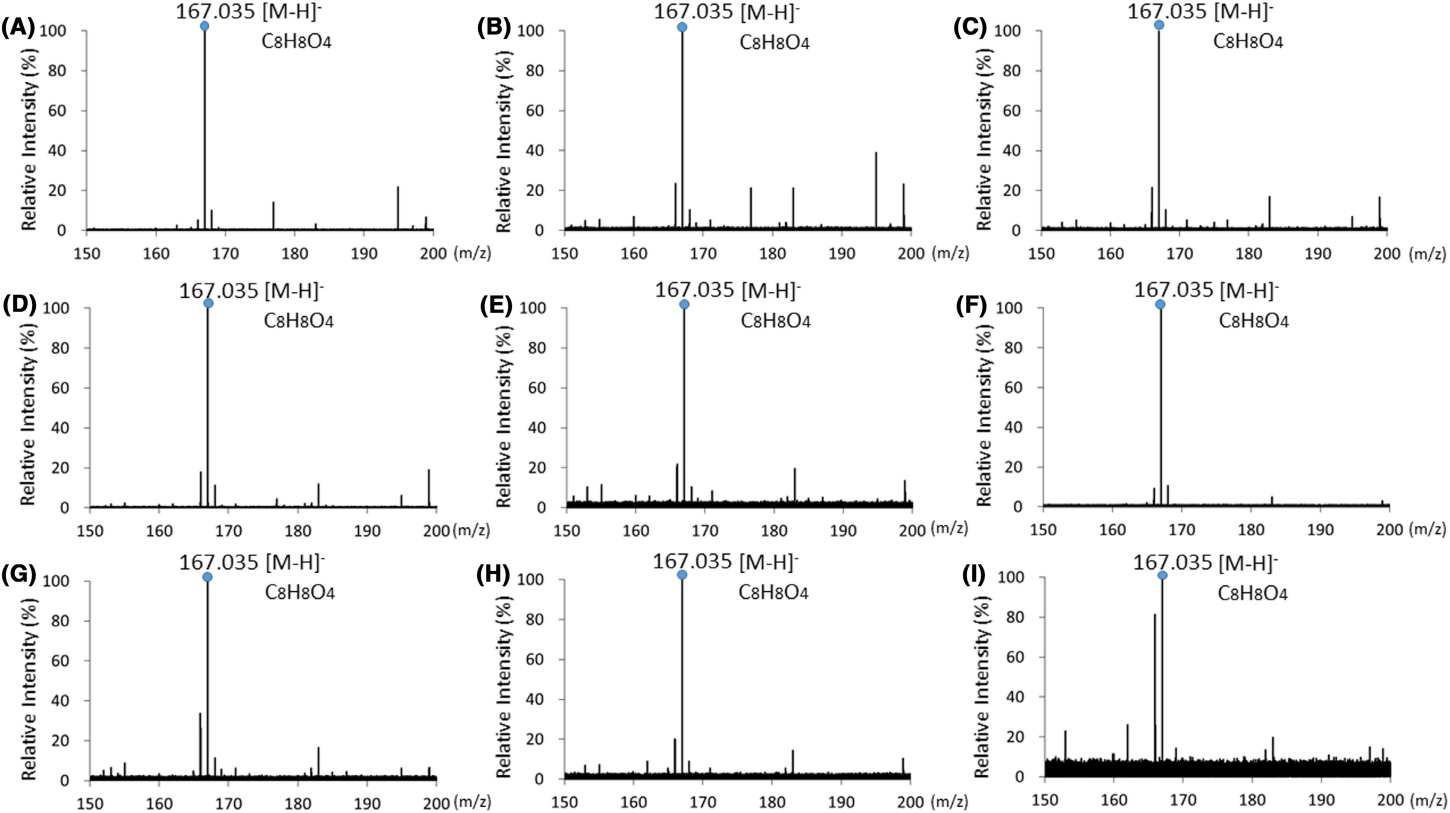
ESI‐MS spectra of HGA solutions. (A) HGA solution at pH 6.0. (B) HGA solution at pH 6.0 after incubation at room temperature for 24 h. (C) HGA solution at pH 6.0 after incubation with KOH at room temperature for 1 h. (D) HGA solution at pH 7.0. (E) HGA solution at pH 7.0 after incubation at room temperature for 24 h. (F) HGA solution at pH 7.0 after incubation with KOH at room temperature for 1 h. (G) HGA solution at pH 8.0. (H) HGA solution at pH 8.0 after incubation at room temperature for 24 h. (I) HGA solution at pH 8.0 after incubation with KOH at room temperature for 1 h.

We analyzed the chemical reaction of HGA (C_8_H_8_O_4_ M.W. 168.15) solutions by ESI‐MS (Figure 4). The HGA solution at pH 6.0 showed a deprotonated molecular ion peak [M–H]^−^ at m/z 167.035 in the negative ion mode (Figure 4A), and after incubation at room temperature for 24 h, it still showed an ion peak at m/z 167.035 [M–H]^−^ (Figure 4B). Furthermore, the HGA solution at pH 6.0 showed the same ion peak at m/z 167.035 [M–H]^−^ after the addition of KOH (Figure 4C). Similar deprotonated molecular ion peaks were obtained for the HGA solutions at pH 7.0 and 8.0 (Figure 4D–I).

**FIGURE 5:**
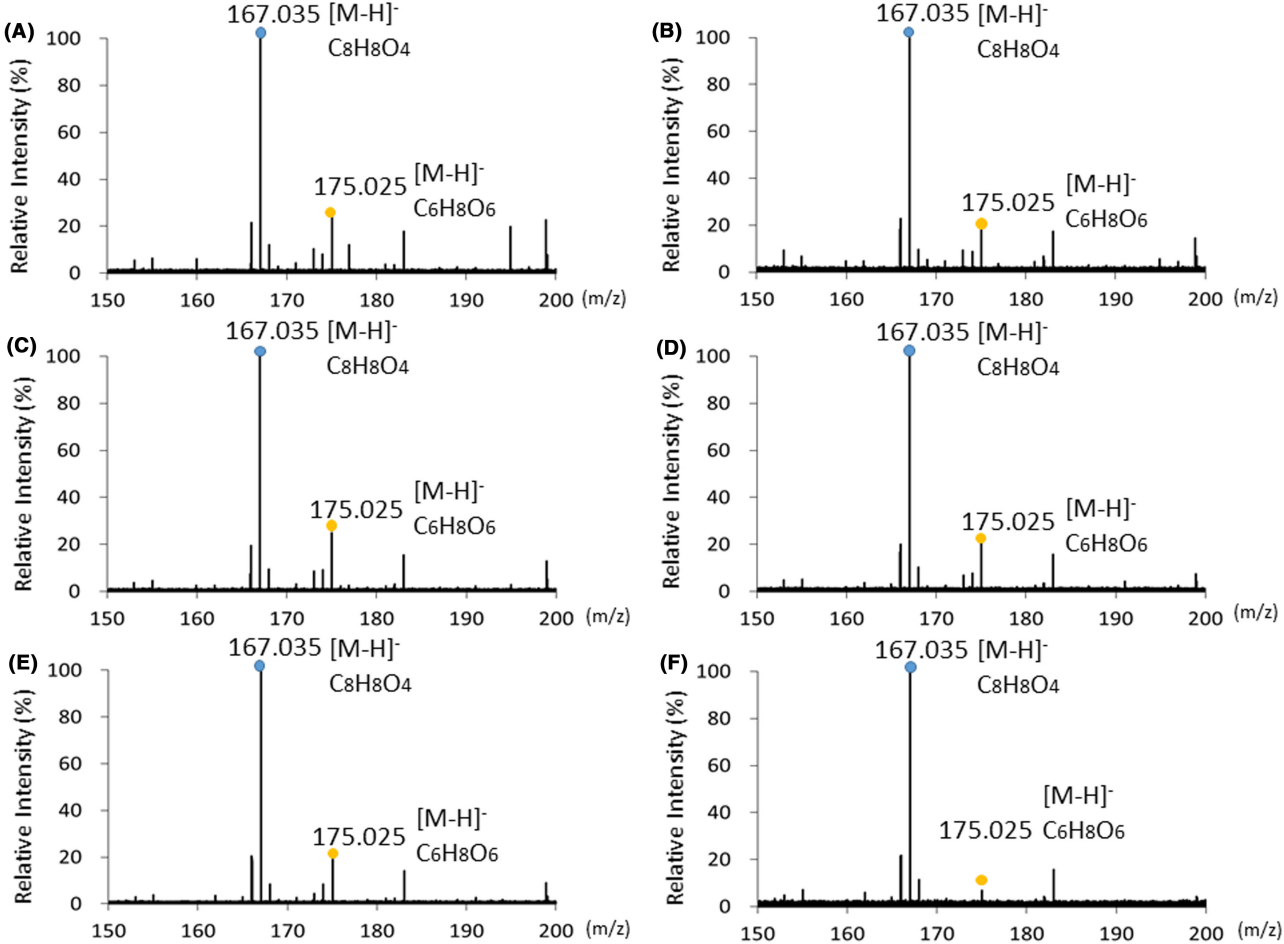
ESI‐MS spectra of HGA solutions treated with AA. (A) HGA solution at pH 6.0 was treated with AA. (B) HGA solution at pH 6.0 was treated with AA after incubation with KOH at room temperature for 1 h. (C) HGA solution at pH 7.0 was treated with AA. (D) HGA solution at pH 7.0 was treated with AA after incubation with KOH at room temperature for 1 h. (E) HGA solution at pH 8.0 was treated with AA. (F) HGA solution at pH 8.0 was treated with AA after incubation with KOH at room temperature for 1 h.

In addition, we analyzed the reaction of HGA solutions treated with AA (C_6_H_8_O_6_, M.W. 176.12) by ESI‐MS (Figure 5). The HGA solution at pH 6.0 treated with AA exhibited the deprotonated molecular peak [M–H]^−^ of HGA at m/z 167.035 and that of AA at m/z 175.025 (Figure 5A). The HGA solution at pH 6.0 treated with AA after the addition of KOH also exhibited the deprotonated molecular ion peak [M–H]^−^ of HGA at m/z 167.035 and that of AA at m/z 175.025 (Figure 5B). Similar deprotonated molecular ions were obtained for the HGA solutions at pH 7.0 and 8.0 treated with AA (Figure 5C–F).

## 4 DISCUSSION

In the present study, we observed the changes in the color and mass spectra of HGA solutions. The deprotonated molecular ion peaks of HGA were detected in all sample solutions by ESI‐MS, regardless of different pH conditions (pH 6.0, 7.0, and 8.0), color changes, or the presence of AA. These results may contribute to the development of HGA detection methods in urine samples obtained from patients with alkaptonuria.

Previous studies reported that HGA consumed more oxygen under alkaline conditions.^8^, ^9^ Therefore, we examined the color change of the HGA solution at pH 6.0, 7.0, and 8.0. Although the HGA solutions under acidic and neutral conditions (pH 6.0 and 7.0) showed no color change, the HGA solution under the alkaline condition (pH 8.0) turned dark brown after incubation at room temperature for 24 h. This color change is likely caused by the oxidation of HGA to benzoquinone acetic acid (BQA).^7^, ^13^ We have also reported that the HGA solution and alkaptonuric urine became dark brown after the addition of an alkali, owing to the oxidation of HGA to BQA.^11^ Herein, we observed the color changes of HGA solutions under different pH conditions (pH 6.0, 7.0, and 8.0) after the addition of KOH, a strong alkali. The HGA solution at pH 8.0 became darker brown following the addition of KOH. In contrast, the HGA solutions at pH 6.0 and 7.0 did not show color changes. Notably, the pH of urine is influenced by daily diet.^14^, ^15^ Based on these results, when the urinary pH of patients with alkaptonuria is less than 7 (from neutral to acidic condition), it may be difficult to observe the brown color change, even if an alkaline solution is added to the urine. Therefore, although the dark brown color change is characteristic of alkaptonuric urine, which contains HGA in excessive amounts,^5^ it is important to consider the pH and contaminants of the urine in confirming the color change.

We have previously reported that AA, an antioxidant, inhibited the oxidation of HGA to BQA in alkaptonuric urine and HGA solutions.^11^, ^12^ In the present study, we analyzed the HGA solutions treated with AA. All HGA solutions (from pH 6.0 to 8.0) treated with AA did not exhibit remarkable color changes, and the HGA solution at pH 8.0 treated with AA showed a weaker brown color after the addition of KOH than that of the HGA solution at pH 8.0 without AA. AA, which is known as vitamin C, is included in many foods and drinks, and urine that contains AA may cause false‐negative results for urinalysis of glucose, blood, nitrite, and bilirubin.^16^, ^17^ Thus, despite the addition of KOH to alkaptonuric urine, the patients with alkaptonuria taking high doses of AA may yield no color change in the urine.

Our results indicated that it might be difficult to observe the dark brown color change of alkaptonuric urine containing HGA because of the influences of pH and AA. Therefore, we further analyzed the reaction of HGA solutions by ESI‐MS. Although the HGA solutions exhibited different color changes in a pH‐dependent manner and oxidation of the HGA solution at pH 8.0 after the addition of KOH was inhibited by AA, ESI‐MS analysis detected the deprotonated molecular ion peak of HGA at m/z 167.035 in all HGA solutions. Moreover, ESI‐MS analysis detected not only HGA but also the deprotonated molecular ion peak of AA at m/z 175.025. These results reveal that ESI‐MS analysis is more useful for detecting HGA in urine than the observation of color change, regardless of the pH or influence of AA.

The HGA solution at pH 8.0 turned brown after incubation at room temperature and turned darker brown after the addition of KOH. However, we could not detect the molecular ion of BQA (C_8_H_6_O_4_), an oxidant of HGA, by ESI‐MS. This may be because HGA is first oxidized to BQA and then to melanin‐like pigments and more complex polymers.^7^, ^13^, ^18^ Therefore, the measurement of HGA by mass spectrometry is more suitable for the detection of alkaptonuric urine than BQA. Moreover, the urine from a patient with alkaptonuria contains excessive amounts of HGA, between approximately 1.8 and 3.5 g/L.^19^ In the present study, we prepared 1.0 g/L HGA solutions and detected the deprotonated molecular ion of HGA in all solutions by ESI‐MS, indicating that the peak appearing at m/z 167.035 is a robust indicator in the screening of alkaptonuria.

In summary, although the color change of HGA solutions was influenced by pH and AA, we identified the molecular ion of HGA in all sample solutions by ESI‐MS, regardless of pH conditions, color changes, or the presence of AA. These results suggest that spectral analysis by ESI‐MS is useful for the detection of HGA and the diagnosis of alkaptonuria.

## FUNDING INFORMATION

This work was supported by JSPS KAKENHI Grant Number JP21K07394.

## CONFLICT OF INTEREST STATEMENT

The authors declare no competing interests.

## Data Availability

The data that support the findings of this study are available from the corresponding author upon reasonable request.
